# Oxidative Stress Biomarkers: Establishment of Reference Values for Isoprostanes, AOPP, and NPBI in Cord Blood

**DOI:** 10.1155/2017/1758432

**Published:** 2017-04-23

**Authors:** Mariangela Longini, Elisa Belvisi, Fabrizio Proietti, Francesco Bazzini, Giuseppe Buonocore, Serafina Perrone

**Affiliations:** ^1^Department of Molecular and Developmental Medicine, University of Siena, Siena, Italy; ^2^UOC Clinical Pathology AOU Senese, Siena, Italy

## Abstract

Oxidative stress (OS) is a common pathogenic factor involved in the onset of several diseases in humans, from immunologic disorders to malignancy, being a serious public health problem. In perinatal period, OS has been associated with adverse outcome of pregnancy and neonatal diseases. Dangerous effects of OS are mediated by increased production of free radicals (FRs) following various mechanisms, such as hypoxia, ischemia reperfusion, hyperoxia, inflammation, mitochondrial dysfunction, Fenton chemistry, and prostaglandin metabolism. FRs have short half-life, and their measurement in vivo is faced with many challenges. However, oxyradical derivatives are stable and thus may be measured and monitored repeatedly. The quantification of OS is based on the measurement of specific biomarkers in biologic fluids and tissues, which reflect induced oxidative damage to lipids, proteins, and DNA. Prostanoids, non–protein-bound iron (NPBI), and advanced oxidation protein products (AOPP) are actually considered truly specific and reliable for neonatal injury. Defining reference values for these biomarkers is necessary to investigate their role in neonatal diseases or also to evaluate the success of treatments. In this work, we wanted to define laboratory reference values for biomarkers of OS in a healthy population of term newborns.

## 1. Introduction

Oxidative stress (OS) has been defined “a state where oxidative forces exceed the antioxidant systems due to loss of the balance between them” [1]. OS reflects the tissue damage resulting from an imbalance between excessive generation of oxidant compounds and insufficient antioxidant defense mechanisms [2]. Oxidant compounds are extremely reactive species capable of independent existence that contains one or more unpaired electron, named free radicals (FRs). They are either endogenous and/or exogenous [1, 3]. Because of their high reactivity, they can abstract electrons from other compounds to obtain stability. Thus, the attacked molecule loses its electron and becomes a FR itself, beginning a chain-reaction cascade, which finally damages the organism's structure and functions. OS is well known to be involved in the pathogenesis of lifestyle-related diseases, including hypertension, diabetes mellitus, ischemic diseases, malignancies, or Alzheimer's disease, Parkinson's disease, and amyotrophic lateral sclerosis [1, 3, 4]. Oxidative compounds are also physiologically relevant in inflammation and tissue repair processes. Hence, they represent some defense mechanisms against microorganisms and malignant cells as well as tissue healing and remodeling [4]. OS is known to be harmful because of the FR that attacks biological molecules, like lipids or proteins, and also DNA. Still, OS has also a useful role in physiologic adaptation and in the regulation of intracellular signal transduction [5]. Oxidative damage has been identified in the pathogenesis of many preterm newborn diseases, such as retinopathy of prematurity (ROP), bronchopulmonary dysplasia (BPD), necrotizing enterocolitis (NEC), patent ductus arteriosus (PDA), periventricular leukomalacia (PVL), and intraventricular hemorrhage (IVH) [6–11]. Hypoxia, hyperoxia, ischemia, and inflammation are main mechanisms of FR overproduction [12–19]. After the occurrence of hypoxia-ischemia, iron ions, serving as transition metal molecules catalyzing hydroxyl radical production via the Fenton reaction and the Haber-Weiss cycle, accumulate in cells. Iron and FRs may result in DNA strand breaks [20], protein and lipid peroxidation [21], and cellular inflammation and death [22, 23].

The accurate measurement of OS in vivo is necessary to investigate their role in lifestyle diseases or also to evaluate the success of treatment. FRs have very short half-life (of the order of few seconds), and their measurement in vivo is faced with many challenges. However, oxyradical derivatives (e.g., hydrogen peroxide or lipid hydroperoxides) are stable and have long half-life (hours to weeks) and thus may be measured and monitored repeatedly.

The quantification of OS is based on the measurement of specific biomarkers in biologic fluids and tissues, which reflect induced oxidative damage to lipids, proteins, and DNA or an increased risk for injury to macromolecules. Several biomarkers have been proposed for OS detection, but only a small number of them can be considered truly specific and reliable for OS injury; these include prostanoids, non–protein-bound iron (NPBI), and advanced oxidation protein products (AOPP) [11, 24, 25].

### 1.1. Prostanoids

Prostanoids are a family of lipid mediators generated by the action of cyclooxygenase on long-chain unsaturated fatty acids. The mechanism involved in their formation implies that FR insult causes hydrogen abstraction from arachidonic acid and addition of molecular oxygen to form a peroxyl radical [26]. The following intermediates undergo double 5-exo-trig cyclization and addition of second molecular oxygen to form prostaglandin G2-like compounds, which are rapidly reduced to F2-IsoPs [27, 28]. These prostanoids are more stable compared with other peroxidation products, such as aldehydes or peroxyl radicals; thus, they can be detected in biologic fluids [29]. Prostanoids can be measured in plasma, tissues, cells, urine, cerebral spinal fluid, bile, and bronchoalveolar lavage fluid [30] for the assessment of in situ oxidative injury. F2-IsoP detection and measurement requires sophisticated and expensive methods, such as liquid chromatography/mass spectrometry. IsoPs are chemically stable in vitro and in vivo and are specific and reliable markers of lipid peroxidation. They are thus reliable markers of in situ oxidative injury [30].

### 1.2. Non–Protein-Bound Iron (NPBI)

In physiologic conditions, iron is safely sequestered by transport proteins, such as transferrin and lactoferrin, and stored in proteins, such as ferritin and hemosiderin [31].

Because iron ions cannot be free in plasma, the term NPBI was introduced to indicate a low-molecular-mass iron form, free from binding to plasma proteins. NPBI levels can be measured using high-performance liquid chromatography [32]. Iron toxicity is inversely proportional to the presence of ferritin, which is able to bind and detoxify ferrous ion, and directly proportional to the quantity of hydrogen peroxide to produce hydroxyl radicals through the Fenton reaction. Furthermore, lipid exposure to high concentration of NPBI leads to the formation of IsoPs.

Non–protein-bound iron is a marker of potential OS because it indicates increased susceptibility to oxidative damage especially in in vivo studies [24].

### 1.3. Advanced Oxidation Protein Products (AOPP)

AOPP is a very important biomarker of OS because the proteins are the major targets of FRs, being present and abundant in cells, plasma, and most tissues [11]. It was severally reported that AOPP level increases in hypoxic newborns, especially preterm babies [25, 33]. Radical-induced damage to proteins is not the terminal process of a reaction but is an enhancer of tissue damages, very common in preterm babies. AOPP remain stable during sample storage both at −20 and at −80°C for six months, allowing for batched analysis of progressive specimens [11]. AOPP are measured using spectrophotometry on a microplate reader. The instruments were calibrated with chloramine-T solutions that absorb at 340 nm in the presence of potassium iodide.

RIs of the OS biomarkers in cord blood are important for screening, diagnosis, and monitoring of perinatal diseases. Reference values for these biomarkers are currently lacking.

The aim of this study is to produce the reference intervals (RIs) for OS markers in cord blood.

## 2. Materials and Methods

The study was conducted in 120 term newborns (58 males and 62 females), with a gestational age (GA) between 38 and 42 weeks, born from vaginal delivery in Siena, Policlinico le Scotte, AOU Senese, Italy, from 01/01/2016 to 30/04/2016. None of the infants required medical care. Newborns with clinical signs of hypoxia ischemia, infection, major congenital malformations, inborn errors of metabolism, and blood group incompatibility were excluded from the study. Birth weight was adequate for gestational age in all enrolled newborns. 120 samples of cord blood for F2-IsoPs, AOPP, and NPBI were examined. The treatment of the sample was previously standardized according to internal protocols of the laboratory. The cordo blood was collected in serum tubes (Sarstedt, S-Monovette Serum gel) and centrifuged promptly after collection, and serum aliquots were preserved at −80°C in Sarstedt Eppendorf Type D (CLSI/NCCLS document H18) [34].

In some aliquots (for the assay of F2-IsoPs), BHT (butylated hydroxytoluene) was added to inhibit the lipidic peroxidation in vitro. All samples were collected within four months.

Inclusion criteria were applied in the second days of life evaluating the clinical conditions and the history of the neonate.

The number of enrolled cases was decided following IFCC guidelines. The Clinical Laboratory Standards Institute (CLSI) recommends a minimum of 120 individuals; this is the minimum sample size required to determine 90% confidence intervals (CI) for the 95th percentile reference limits (2.5th and 97.5th percentiles).

The preanalytical phase has been standardized: the permanence time in the freezer is not higher than four months; for these reasons, we have chosen to enlist a smaller number of cases, but certainly more homogeneous.

The LC-MS/MS method of Casetta et al. [30] was followed for determination of F2-IsoPs (API 4000 Tandem Mass Spectrometer coupled with HPLC Agilent 1200 series), the method of Paffetti et al. [24] for NPBI with HPLC-DAD system (Agilent 1100 series), and the spectrophotometric method of Witko-Sarsat et al. [35] for AOPP detection.

The methods that we used describe the analytical imprecision, the limit of detection, the linearity, the recovery, the interference characteristic, and the traceability of the results. Hemolytic samples were excluded. All samples for each method were measured in double, in three different days in the same conditions, with the same lot and technologist variability, from the same people. Previously, tests on the storage stability were made.

## 3. Statistical Analysis

We used an “a posteriori” approach.

The indirect method of sampling suggested by Horn and Pesce [36] was used to estimate IRs for F2-IsoP, NPBI, and AOPP in cord blood sample of newborn.

The descriptive statistical analysis, after the D'Agostino-Pearson test for normality of population was performed, included median and IQR. The RIs were calculated using a nonparametric method as described in the CLSI guidelines C28-A3. The IFCC recommends that a minimum number of subjects of 120 should be recruited to derive RIs.

Statistics were performed using SPSS version 20 (IBM Corporation, NY, USA).

## 4. Results

Serum samples from 120 umbilical cords were used to calculate reference RIs for 3 specific markers of OS measured with chromatography (HPLC), liquid chromatography/mass spectrometry (LC/MS/MS), or spectrophotometry methods.

All infants were from a normal pregnancy ended in a spontaneous delivery. During pregnancy, the mothers followed the same living and eating style.

Demographic and clinical characteristics of the study population are reported in Table 1.

Sample size, median, 25 and 75 percentiles for OS markers in cord blood, and *P* values computed by the D'Agostino-Pearson test for normality (Sheskin, 2011) are detailed in Table 2.

RIs, 90% CI, and medians are calculated with a right side nonparametric method (CLSI C28-A3) for OS markers in cord blood and are provided in Table 3.

Our data showed a “right sided” distribution with only an upper limit of reference and no lower limit. Outliers have been removed using the Tukey test.

**Figure 1:**
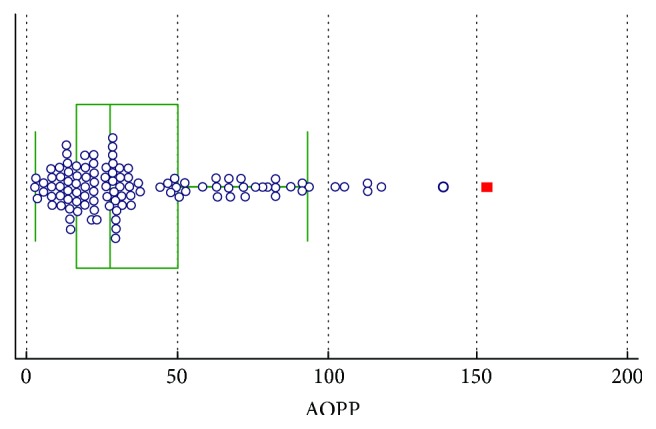
Box and whisker plot for AOPP.

**Figure 2:**
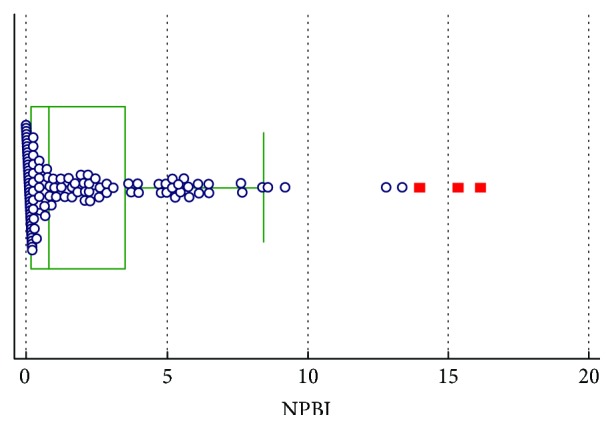
Box and whisker plot for NPBI.

**Figure 3:**
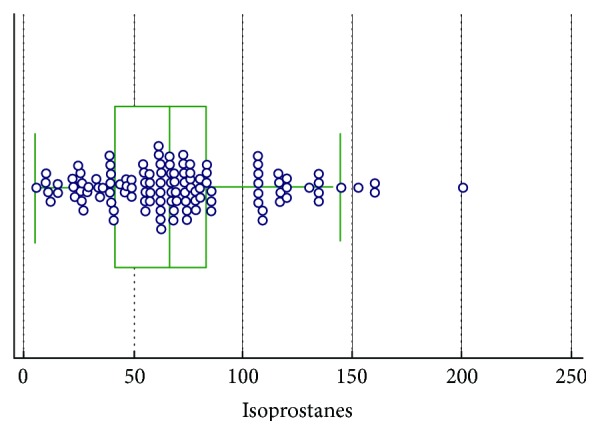
Box and whisker plot for isoprostanes.

In Figures 1–3, groups of numerical data through their quartiles are graphically depicted (box and whisker plot). Outliers are plotted as filled points.

## 5. Discussion

Our study was born from the need to provide reference values for markers of OS.

Clinical laboratory data are not used if they cannot be related with the own RIs. The value of a laboratory data is helpful only if it is compared with the RIs.

The RIs can be a single cut, a series of cut-offs, or a range of values containing 95% of the results of a reference population. There are more possibilities to compare the laboratory data: the most used are the reference collective values. These are essential for screening, detection, and monitoring of diseases. They are the most used but are not easy to produce.

They should be determined on a representative sample of the patient population where the test will be used. The most common definition of the RIs is the range of values containing the central 95% of the healthy population. If the reference limits are the values at 2.5% and 97.5%, the other 5% of the “healthy” population is to be classified as “abnormal” or “positive”.

These data allow you to compare the values of a patient with the reference data of the reference population, but they have no value for the medical decision levels.

We decided to measure F2-isoprostanes, non–protein-bound iron (NPBI), and advanced oxidation protein products (AOPP) as markers of OS damage since they are considered truly specific and reliable to evaluate the OS damage. Moreover, we used methodologies with a high sensitivity and specificity.

The RIs were produced by following safe guidelines edited by the Clinical and Laboratory Standard Institute used for the making of sample collection, the process of analysis, and statistical processing.

Specific guidelines related to production protocol are proposed for establishing RIs. These procedures include the choice of the preanalytical and analytical phases, the calculation methods, and the requirements for estimating valid reference intervals [37–40].

Ideally, RIs should be determined by sampling a healthy population using a direct sampling method (“a priori”). The direct technique conforms to the International Federation of Clinical Chemistry and Laboratory Medicine (IFCC) recommendations, and it is preferred. However, the particularity of populations and the time involved in obtaining a representative group of reference individuals may be overcome with the indirect method. The working groups acknowledge that, in this circumstance, it may be very difficult to have a priori method and they advocate the use of indirect methods in which specific statistical tests for small populations are applied [41–43].

In a posteriori method, all the processes (exclusion and partition of reference individuals) occur after a biological sample is collected and analyzed.

It involves application of statistical methods to analytical values collected in an already made database without previous choice of reference individuals. The indirect technique may have clinical utility in selected situations, including pediatrics and the elderly population where collecting a sufficient numbers of reference samples may be difficult [41–43].

This method is based on the concept that many results, even on hospital patients, may be “normal.” Many studies used data from all hospital patients to produce reference values. In this study, we enrolled neonates that were born healthy at the end of a physiological pregnancy and were discharged from hospital in the second days of life.

The production of reference values seems to be quick and easy, but it is quite a laborious process as reported in the lines of the expert group on the reference value theory (EPTRV) of the International Federation of Clinical Chemistry and Laboratory Medicine (IFCC) and in the Standing Committee on the reference values—International Council for the standardization of procedures hematology (ICSH) [37].

In our study, the guidelines for the production of reference values have been produced by using the C28-A2 of the National Committee for Clinical Laboratory Standards (NCCLS).

We choose a representative group of the population that will be tested. The reference group should be free from disease and conditions that could lead to an “abnormal” result. We established criteria to exclude individuals with factors that can affect the test.

Reed et al. [42] suggest that a minimum of 120 observations, each one from a referent subject, should be available for analysis. This has the advantage of also allowing 90% confidence limits to be computed nonparametrically for each reference limit.

To estimate the reference limits for these same percentiles with 95% confidence, a minimum of 146 reference values are needed; for 99% confidence, a minimum of 210 reference values are needed. Linnet [43] proposes that up to 700 should be obtained for highly skewed distributions of results. However, as a standard for general practice, the working group supports the recommended minimum of 120 reference subjects. When fewer observations are available, the use of the nonparametric method becomes problematic. The robust method, however, offers a potential alternative.

As noted by Horn and Pesce [36], calculating the reference interval using robust methods involves an iterative process, where the initial location (center) is estimated by the median and the initial stairs (spread) and by the median absolute deviation about the median (MAD). In the process, actual observations are down weighted according to their distance from the central tendency of the sample. The quantity that represents the updated estimate of central tendency is calculated for each iteration, until the change in successive iterative values is negligible.

The decision to choose 120 cases is due to a desire to follow, for our small population, the ICSH guidelines that regulate the preanalytical phase and the analysis phase of the treatment of samples. To recruit a greater number of cases, we would have to wait more time; this could have led to an increase in the error in the preanalytical and in the analytical phases.

When it is not possible to reach the suggested number of cases, Horn and Pesce [36] have proposed the robust method as an alternative to estimate reference RIs. However, we discarded this possibility because the CI intervals are estimated with the bootstrap [44] by using this method. When the sample contains too many same values, it may be impossible to calculate the CI. For NPBI, zero concentration values were reported many times and the CI was not calculated.

In conclusion, our study for the first time in literature provides reference value for the most reliable markers of OS in newborns. These intervals are necessary for all clinical laboratory tests, and they are an important task for producing diagnostic tests for clinical pathology.

## Figures and Tables

**Table 1:** Demographic and clinical characteristics of the study population.

*N*	120
M	58
F	62
Enrollment	1/01/2016–30/04/2016
Maternal age	30,08 ± 3,19
GA (wks)	39,08 ± 1,24
BW (g)	3247,03 ± 495,5
pH	7 ± 0,12
Apgar	9-10

*N*: sample number; M: male; F: female; GA: gestational age; BW: birth weight.

**Table 2:** Summary statistic for oxidative stress markers in cord blood.

	Sample size	Median	Percentiles (25°–75°)	*P* (D'Agostino Pearson)
AOPP	120	27.90	16.40–50.50	<0.0001
NPBI	120	0.80	0.2–3.53	<0.0001
Isoprostanes (pg/mL)	120	66.30	41.02–83.70	<0.0001

**Table 3:** Reference interval—right side nonparametric method (CLSI C28-A3) for OS markers in cord blood.

Analytes	Upper limit	Median	Higher 90% CI (bootstrap^a^ CI)
AOPP (*μ*mol/dL)	80.39	27.90	68.94–92.28
NPBI (*μ*mol/L)	6.91	0.80	5.48–8.30
Isoprostanes (pg/mL)	124.47	66.30	114.31–136.58

^a^Bootstrap confidence interval (500 iterations; random number seed: 400).
